# Should Cancer Survivors Fear Radiation-Induced Sarcomas?

**DOI:** 10.1080/13577149778407

**Published:** 1997-06

**Authors:** Malcolm Feigen

**Affiliations:** The Radiotherapy Centre Austin & Repatriation Medical Centre Repatriation Campus Locked Bag 1 Heidelberg West Victoria 3081 Australia


*Sarcoma* (1997) 1, 113–114

LETTER TO THE EDITOR

### Should cancer survivors fear radiation-induced sarcomas?

*Sir*: We read with interest the leading article in your inaugural *Sarcoma* issue on the subject of radiotherapy-induced sarcomas,[1] and congratulate the author on a meticulous review of the literature. We do, however, have some additional comments to make with respect to this important subject.

First, while we would certainly agree that the absolute incidence of radiotherapy-induced sarcomas is low—and that this in itself should not suffice to deter patients from receiving radiotherapy where indicated—it is clear that sarcomas represent only a minority of all radiation-induced neoplasms.[2] Accordingly, readers of the review should not mistakenly infer that the risks of radiation-induced neoplasms in general are necessarily negligible or trivial.

Second, it is stated that improved radiotherapy techniques over the past two decades deliver less damage to surrounding tissues, and hence can be assumed to be less carcinogenic. Although this is a popular assumption, the well-recognized biphasic relationship between radiation dose and tumour induction cautions that it may not necessarily prove to be correct.[3] Indeed, our own observations have suggested that second tumours tend to cluster around the field edge, rather than within the most 'damaged' region of irradiation (unpublished data). Techniques to minimize beam scatter may therefore remain relevant despite other modern technical improvements.[4]

Third, certain patient subsets may be at significantly higher risk of radiogenic second malignancy than others, and hence less appropriately reassured by the findings of the review. In particular, we suggest that young patients ($<35$ years) fall into this category[3,4] and advise that these patients and their oncologists continue to include the hazard of second malignancy in their therapeutic decision-making algorithm.

RICHARD EPSTEIN MD PhD FRCP
*Division of Medicine, Imperial College School of Medicine, Charing Cross Hospital, Fulham Palace Road, London W6 8RF, UK*

IAIN HANHAM FRCR FRCP
*Department of Radiotherapy, Charing Cross Hospital, Fulham Palace Road, London W6 8RF, UK*

ROGER DALE PhD FIPEM
*Department of Radiation Physics & Radiobiology, Charing Cross Hospital, Fulham Palace Road, London W6 8RF, UK*

### References

1 Feigen M. Should cancer survivors fear radiation-induced sarcomas? *Sarcoma* 1997; 1:5–15.
2 United Nations Scientific Committee on the Effects of Atomic Radiation. Sources and effects of ionizing radiation: report to the General Assembly with scientific annexes. *United Nations Sales Publication E.94.IX.11.* United Nations: New York, 1994.
3 Epstein R, Hanham I, Dale R. Radiotherapy-induced second cancers: are we doing enough to protect young patients? *Eur J Cancer* 1997; 33:526–30.
4 Epstein RJ, Kelly SA, Cook M, *et al.* Active minimisation of radiation scatter during breast radiotherapy: management implications for young patients with good-prognosis primary neoplasms. *Radiother Oncol* 1996; 40:69–74.

*Sir*: I wish to dispute the comments on my article by Epstein *et al.* regarding the occurrence of second tumours in the reduced dose regions at radiation field edges. There is much evidence to the contrary, from the development of breast cancers in mantle ports of women treated for Hodgkin's disease[1,2] to the reports of sarcomas in regions of field overlap.[3,4] In support of their hypothesis, Epstein *et al.* quote

1357-714X/97/020113-02 $9.00 ©1997 Carfax Publishing Ltd

Karlsson *et al.* in their work,[3,4] but even including cases of Stewart–Treves syndrome, 13 out of 17 cases of soft tissue sarcomas in this report[5] are inside radiation fields, and integral dose was the most significant correlating factor. No evidence of an increased risk for non-breast malignancies in the vicinity of irradiated fields was reported in the analysis of 1382 autopsies of breast cancer patients treated at Roswell Park, where 86% of patients had breast cancer as their only malignancy at the time of death.[6]

MALCOLM FEIGEN
*The Radiotherapy Centre, Austin & Repatriation Medical Centre, Repatriation Campus, Locked Bag 1, Heidelberg West, Victoria 3081, Australia*

### References

1 Hancock SL, Tucker MA, Hoppe RT. Breast cancer after treatment of Hodgkin's disease. *J Natl Cancer Inst* 1993; 85:25–31.
2 Tinger A, Wasserman TH, Klein EE, *et al.* The incidence of breast cancer following mantle field radiation therapy as a function of dose and technique. *Int J Radiat Oncol Biol Phys* 1997; 37:865–70.
3 Pierce SM, Recht A. Lingos TI, *et al.* Long-term radiation complications following conservative surgery and radiation treatment for patients with early stage breast cancer. *Int J Radiat Oncol Biol Phys* 1992; 23:915–23.
4 Bobin JY, Rivoire M, Delay E, *et al.* Radiation-induced sarcomas following treatment for breast cancer: presentation of a series of 14 cases treated with an aggressive surgical approach. *J Surg Oncol* 1993; 2:175–85.
5 Karlsson P, Holmberg E, Johansson KA, *et al.* Soft tissue sarcoma after treatment of breast cancer. *Radiother Oncol* 1996; 38:25–31.
6 Mamounas EP, Perez-Mesa C, Penetrante RB, *et al.* Patterns of occurrence of second primary non-mammary malignancies in breast cancer patients: results from 1382 consecutive autopsies. *Surg Oncol* 1993; 2:171–85.
